# Chronic obstructive pulmonary disease is associated with an increased risk of herpes zoster: A retrospective United States claims database analysis

**DOI:** 10.1111/crj.13554

**Published:** 2022-11-22

**Authors:** Philippe Thompson‐Leduc, Parinaz Ghaswalla, Wendy Y. Cheng, Min‐Jung Wang, Michael Bogart, Brandon J. Patterson, Mei Sheng Duh, Suna Park, Barbara P. Yawn

**Affiliations:** ^1^ Health Economics and Outcomes Research Analysis Group, Inc. Montreal Quebec Canada; ^2^ US Health Outcomes and Epidemiology – Vaccines, GSK Philadelphia Pennsylvania USA; ^3^ Health Economics and Outcomes Research Analysis Group, Inc Boston Massachusetts USA; ^4^ Value Evidence and Outcomes, GSK Research Triangle Park North Carolina USA; ^5^ Department of Family Medicine and Community Health University of Minnesota Minneapolis Minnesota USA; ^6^ Present address: Health Economics and Outcomes Research Moderna Cambridge Massachusetts USA; ^7^ Present address: Amgen Inc. Thousand Oaks California USA; ^8^ Present address: Patient Engagement, Gilead Sciences Foster City California USA; ^9^ Present address: Global Commercial Strategy Organization Janssen Global Services, LLC Raritan New Jersey USA; ^10^ Present address: Global Evidence and Outcomes – Gastroenterology Data Sciences Institute, R&D, Takeda Pharmaceuticals Cambridge Massachusetts USA

**Keywords:** chronic obstructive pulmonary disease, herpes zoster, incidence rate, United States

## Abstract

Chronic obstructive pulmonary disease (COPD) has been reported as a potential risk factor for developing herpes zoster (HZ). We aimed at comparing incidence rates of HZ between people with versus without COPD in the US. This retrospective cohort study used data from Optum's de‐identified Clinformatics Data Mart database from 1/1/2013 through 12/31/2018. We identified two cohorts of people ≥40 years without prior HZ, HZ vaccination, postherpetic neuralgia (PHN) or HZ ophthalmicus: those with (COPD+) and those without (COPD−) a COPD diagnosis. Adjusted incidence rate ratios (aIRRs) of HZ and PHN were calculated using generalized linear models, controlling for the propensity score of being diagnosed with COPD and relevant demographic and clinical characteristics. People in the COPD+ cohort (*n* = 161 970) were considerably older, had more comorbidities and were more likely to use corticosteroids than those in the COPD− cohort (*n* = 9 643 522). The incidence rate of HZ was 5.7‐fold higher in the COPD+ versus COPD− cohorts (13.0 vs. 2.3 per 1000 person‐years [PY]; aIRR, 2.77; 95% confidence interval [CI], 2.69 to 2.85; *P* < 0.001). The unadjusted incidence rate of PHN was 1.7‐fold higher in the COPD+/HZ+ versus COPD−/HZ+ cohort (64.8 vs. 37.1 per 1000 PY), but not after adjustment (aIRR, 1.07; 95% CI, 0.79 to 1.45). HZ and PHN incidence rates increased with age. After adjustment, COPD+ adults had a 2.8‐fold increased risk of developing HZ. These results may help to increase awareness about potential risk factors for HZ and highlight the need for vaccination among those at increased risk.

## Graphical abstract

This study reviewed 2013–2018 US insurance claims data to compare the incidence rates of herpes zoster among people 40 years and older with and without chronic obstructive pulmonary disease. Adjusting for baseline demographics and clinical characteristics, this study found that people with chronic obstructive pulmonary disease were nearly three times more likely to develop herpes zoster than those without.

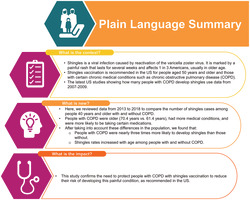

## 1 INTRODUCTION

Herpes zoster (HZ), commonly known as ‘shingles’, results from reactivation of the varicella zoster virus, which remains latent in the sensory ganglia after an earlier varicella infection.^1^ HZ causes a painful rash, often on the trunk, which usually lasts around 2–4 weeks.^2^ Approximately 1 in 3 people in the United States (US) will develop HZ at some point during their life, resulting in around 1 million cases each year.^3^ The incidence rate of HZ increases with age, with estimates of approximately 3.9, 7.5 and 12.0 per 1000 person‐years (PY) for people aged 31–40, 51–60 and ≥71 years, respectively (during 2007–2018 in the US).^4^ The incidence of HZ has also increased over time in the US, doubling from around 2.9 to 5.8 per 1000 PY from 1994 to 2018.^4^ Depending on age, around 5–20% of people with HZ develop postherpetic neuralgia (PHN),^5^ which causes neuropathic pain that can last from 3–4 months to >1 year.^2^

The US Advisory Committee on Immunization Practices (ACIP) first recommended HZ vaccination in 2008, with a live attenuated vaccine (Zoster Vaccine Live [ZVL]) for adults aged ≥60 years.^6^ Following approval of the adjuvanted Recombinant Zoster Vaccine (RZV) in 2017,^7^ the ACIP provided a preferential recommendation for RZV over ZVL for the prevention of HZ and related complications in immunocompetent adults aged ≥50 years.^8^ Despite these recommendations, HZ vaccination coverage among people aged ≥50 years in the US in 2019 was only 26.1%.^9^

**FIGURE 1:**
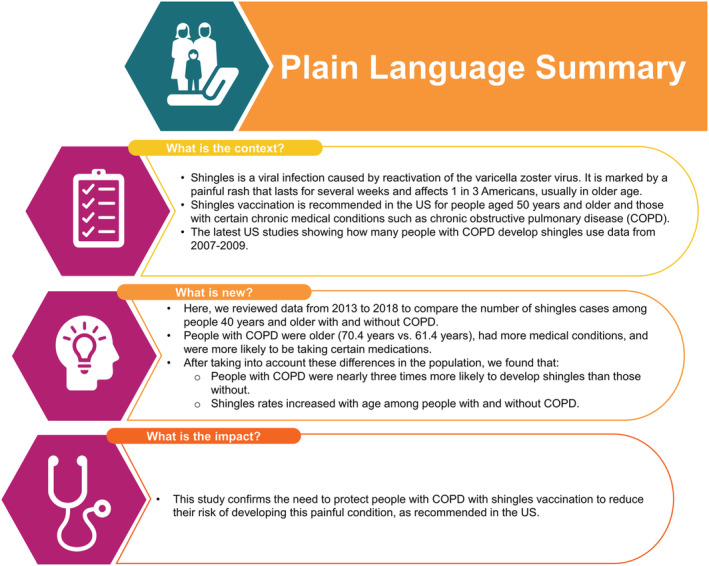
Plain language summary

The ACIP recommendations for HZ vaccination also recommend RZV for adults with certain chronic medical conditions, including chronic pulmonary disease.^8^ Various studies from around the world have reported that the incidence of HZ is 1.1‐ to 1.7‐fold higher among people with versus without chronic obstructive pulmonary disease (COPD).^10^, ^11^, ^12^, ^13^, ^14^, ^15^, ^16^, ^17^ While three of these previous studies are from the US,^11^, ^12^, ^15^ none included recent data. To fill the literature gap in the recent understanding of the burden of HZ in COPD, the objectives of the current study were to estimate and compare the incidence rates of acute HZ between people with and without diagnosed COPD in the US. We also estimated the incidence rates of PHN after HZ among people with and without COPD. Figure 1 contains a summary of the context and impact of the study in a format that can be shared with patients. Video S1 provides a video animation with voice over explaining the study design and results.

## 2 MATERIALS AND METHODS

### 2.1 Data source

This study (GSK study identifiers: HO‐19‐19749/VxHO‐000050) used data from Optum's de‐identified Clinformatics Data Mart database (Optum CDM), which includes US administrative health insurance claims associated with medical services and detailed information on prescription fills. Optum CDM covers approximately 13 million annual lives of adults in all US census regions and includes both commercially insured and Medicare Advantage members. Optum CDM contains >36 months of historical data on patient demographics, dates of eligibility, death, claims for inpatient and outpatient visits, costs of services and laboratory tests and results.

### 2.2 Study design and inclusion criteria

In this retrospective cohort study, we identified two cohorts: those with a COPD diagnosis (COPD+ cohort) and those without a COPD diagnosis (COPD− cohort). Details of the International Classification of Diseases (ICD) codes and definitions of COPD used for this study can be found in Table S1. In the COPD+ cohort, the index date was the later of the first observed COPD diagnosis or 6 months after the start of continuous enrollment. In the COPD− cohort, the index date was assigned as 6 months after the start of continuous enrollment. Patients were required to be aged ≥40 years on the index date; have no claims for HZ vaccines or HZ, PHN or HZ ophthalmicus diagnoses (see Table S1) at any time before the index date; and have ≥6 months of continuous enrollment before the index date and ≥18 months of continuous enrollment after the index date.

### 2.3 Baseline data

Age, sex, race/ethnicity, geographic region and type of insurance plan were assessed on the index date. Asthma (see Table S1), Charlson‐Quan Comorbidity Index (CCI) score and its component conditions (identified using ICD codes),^18^ and use of corticosteroids and immunosuppressants was assessed during the 6 months before the index date.

### 2.4 Follow‐up and outcomes

Patients were followed until an HZ diagnosis (see Table S1) or were censored at the first of: diagnosis of PHN or HZ ophthalmicus, receipt of HZ vaccine, end of continuous enrollment or end of data availability. The incidence rates of HZ in the COPD+ and COPD− cohorts were then estimated, overall and in each age group (40–49, 50–59, 60–69, 70–79 and ≥80 years). We also estimated the incidence rates of PHN after an HZ diagnosis among patients with COPD and HZ (COPD+/HZ+) and those with HZ but without COPD (COPD−/HZ+), overall and in each age group. Of note, the baseline and observation periods for the PHN incidence analysis were different from the HZ incidence analysis. Demographics (including age) were assessed at the incident HZ date and clinical characteristics were assessed during the 6 months prior to the incident HZ date; the observation period was the 18 months following the incident HZ date.

### 2.5 Statistical analyses

Baseline demographic and clinical characteristics are described (as means ± standard deviations [SDs] for continuous variables; frequencies and percentages for categorical variables) for the COPD+ and COPD− cohorts. Due to the large sample size, differences between the two cohorts were assessed using standardized differences rather than *P*‐values. Standardized differences were calculated as detailed in Text S1, with values of 20%, 50% and 80% indicating small, medium and large differences, respectively.^19^

The incidence rates of HZ in the COPD+ and COPD− cohorts were calculated by dividing the number of incident HZ events during the observation period by the patient‐time observed and are reported per 1000 PY. The incidence rates of PHN in the COPD+/HZ+ and COPD−/HZ+ cohorts were calculated similarly.

Adjusted incidence rate ratios (aIRRs) for HZ in the COPD+ versus COPD− cohorts were calculated using generalized linear models assuming a Poisson distribution and log link, accounting for the propensity score of being diagnosed with COPD and relevant demographic characteristics (i.e., age at index, sex, region and insurance type) and clinical characteristics (i.e., CCI score; asthma; and use of chemotherapy, immunosuppressants and corticosteroids [short‐term oral, long‐term oral and inhaled]).

PHN aIRRs were also calculated using generalized linear models, but assuming a negative binomial distribution and log link (due to over‐dispersion), accounting for the propensity score of being diagnosed with COPD and the same demographic characteristics and clinical characteristics as for HZ (but at the time of incident HZ).

Statistical analyses were conducted using the statistical software SAS 9.4 or SAS Enterprise Guide 7.1 (SAS Institute Inc., Cary, NC, USA).

## 3 RESULTS

### 3.1 Study population

**FIGURE 2:**
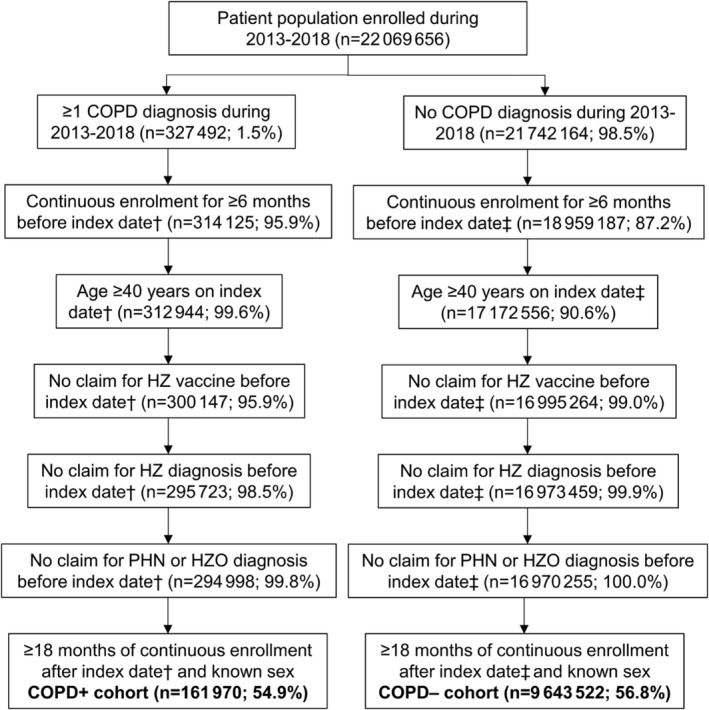
Cohort selection. Abbreviations: COPD, chronic obstructive pulmonary disorder; HZ, herpes zoster; HZO, herpes zoster ophthalmicus; n, number of people; PHN, postherpetic neuralgia. ^†^Assigned as the later of the first observed date of COPD diagnosis or the 6‐month point after the continuous enrollment start date. ^‡^Assigned as the 6‐month point after the continuous enrollment start date

Among 22 069 656 people who were enrolled during 2013–2018, 327 492 received a diagnosis of COPD during this time, while 21 742 164 did not. After applying the inclusion/exclusion criteria, the COPD+ and COPD− cohorts included 161 970 and 9 643 522 people, respectively (Figure 2).

### 3.2 Baseline characteristics

People in the COPD+ cohort were significantly older than those in the COPD− cohort (70.4 ± 10.1 vs. 61.4 ± 12.5 years; Table 1). They were also more likely to have Medicare Advantage coverage and had a higher mean CCI score (Table 1) and more comorbidities, including asthma (Table S2). Those with COPD were also more likely to be using corticosteroids (oral and inhaled) (Table 1).

**TABLE 1:** Demographic and clinical characteristics

	COPD+ cohort (*n* = 161 970)	COPD− cohort (*n* = 9 643 522)	Standardized difference^a^
Age at index date (y), mean ± SD	70.4 ± 10.1	61.4 ± 12.5	78.9%
40–49 y, *n* (%)	5360 (3.3)	2 243 076 (23.3)	58.8%
50–59 y, *n* (%)	21 848 (13.5)	2 285 781 (23.7)	26.3%
60–69 y, *n* (%)	46 871 (28.9)	2 572 484 (26.7)	5.0%
70–79 y, *n* (%)	56 103 (34.6)	1 684 348 (17.5)	39.1%
≥80 y, *n* (%)	31 788 (19.6)	857 833 (8.9)	30.7%
Male, *n* (%)	73 329 (45.3)	4 505 328 (46.7)	2.9%
Race/ethnicity, *n* (%)			
White	105 659 (65.2)	5 698 540 (59.1)	12.7%
Black	15 575 (9.6)	808 027 (8.4)	4.3%
Hispanic	11 094 (6.8)	874 769 (9.1)	8.2%
Asian	2574 (1.6)	309 246 (3.2)	10.6%
Unknown	27 068 (16.7)	1 952 940 (20.3)	9.1%
Geographic region, *n* (%)			
South	65 818 (40.6)	3 916 132 (40.6)	0.1%
West	39 637 (24.5)	2 192 213 (22.7)	4.1%
Midwest	34 813 (21.5)	2 292 920 (23.8)	5.5%
Northeast	20 515 (12.7)	1 098 782 (11.4)	3.9%
Unknown	1187 (0.7)	143 475 (1.5)	7.2%
Insurance type, *n* (%)			
Medicare Advantage	136 879 (84.5)	4 715 557 (48.9)	75.6%
Commercial	25 091 (15.5)	4 927 965 (51.1)	–
Asthma, n (%)	26 297 (16.2)	70 581 (0.7)	55.6%
CCI score,^b^ mean ± SD	1.9 ± 1.9	0.2 ± 0.7	125.7%
Use of oral corticosteroids, *n* (%)	57 384 (35.4)	1 338 991 (13.9)	50.0%
Short‐term use^c^	51 579 (89.9)	1 265 981 (94.5)	17.4%
Long‐term use^d^	5805 (10.1)	73 010 (5.5)	–
Use of inhaled corticosteroids, *n* (%)	48 594 (30.0)	271 907 (2.8)	73.4%
Use of immunosuppressants, *n* (%)	663 (0.4)	26 888 (0.3)	2.2%

Abbreviations: CCI, Charlson‐Quan Comorbidity Index; COPD, chronic obstructive pulmonary disease; *n*, number of patients; SD, standard deviation; y, years.

^a^ Standardized differences of 20%, 50% and 80% suggest small, medium and large differences, respectively.^19^

^b^ Computed according to the methods outlined in Quan et al.^18^ Information on individual CCI conditions can be found in Table S2.

^c^ <6 consecutive weeks.

^d^ ≥6 consecutive weeks, allowing for up to 7 days of gap between two dispensing.

### 3.3 Incidence rates of acute HZ

**FIGURE 3:**
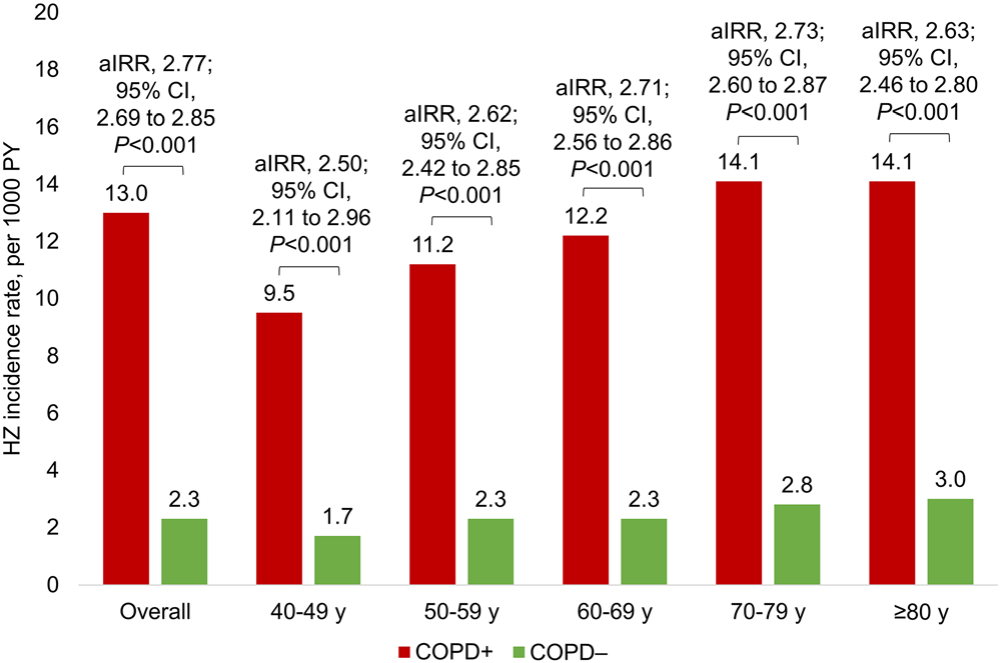
Incidence rates and aIRRs^†^ of acute HZ in the COPD+ versus COPD− cohorts, overall and stratified by patient age at index.^‡,§^ Abbreviations: aIRR, adjusted incidence rate ratio; CCI, Charlson‐Quan Comorbidity Index; CI, confidence interval; COPD, chronic obstructive pulmonary disease; HZ, herpes zoster; *P*, *P*‐value; PY, person‐years; y, years. ^†^aIRRs were calculated using generalized linear models assuming a Poisson distribution and log link, accounting for the propensity score of being diagnosed with COPD and relevant demographic characteristics (i.e., age at index, sex, region and insurance type) and clinical characteristics (i.e., CCI score; asthma; use of chemotherapy, immunosuppressants and corticosteroids [short‐term oral, long‐term oral and inhaled]).^‡^For the numbers of people in each group, please refer to Table 1. ^§^The mean overall observation periods were 36.6 ± 17.2 months in the COPD+ cohort and 37.8 ± 18.3 months in the COPD− cohort, with similar periods by age group

In the COPD+ cohort, there were 6430 HZ diagnoses among 161 970 people during a mean follow‐up of 36.6 months, resulting in an incidence rate of 13.0 per 1000 PY, which was higher than in the COPD− cohort (2.3 per 1000 PY [69 524 HZ cases among 9 643 522 people during a mean follow‐up of 37.8 months]) (Figure 3). After adjusting for demographic and clinical characteristics, the aIRR was 2.77 (95% confidence interval [CI], 2.69 to 2.85; *P* < 0.001). HZ incidence rates increased with age in the COPD+ and COPD− cohorts, from 9.5 to 14.1 per 1000 PY in the COPD+ cohort and from 1.7 to 3.0 per 1000 PY in the COPD− cohort for age groups of 40–49 through ≥80 years (Figure 3). The aIRRs ranged from 2.50 (95% CI, 2.11 to 2.96) among those aged 40–49 years to 2.73 (2.60 to 2.87) among those aged 70–79 years.

### 3.4 Incidence rates of PHN among people with HZ

The baseline characteristics of those who developed HZ during follow‐up are shown in Table S3, by COPD status. Those in the COPD+/HZ+ versus COPD−/HZ+ cohorts were older (73.0 ± 9.6 vs. 65.9 ± 12.5 years), more likely to have an asthma diagnosis, had a higher mean CCI score and were more likely to use corticosteroids (particularly inhaled).

**FIGURE 4:**
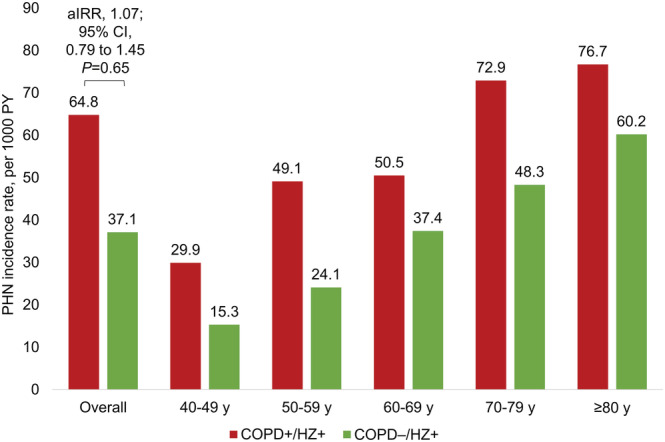
Incidence rates and overall aIRR^†^ of PHN among patients in the COPD+/HZ+ versus COPD−/HZ+ cohorts, overall and stratified by patient age at incident HZ.^‡,§^ Abbreviations: aIRR, adjusted incidence rate ratio; CCI, Charlson‐Quan Comorbidity Index; CI, confidence interval; COPD, chronic obstructive pulmonary disease; HZ, herpes zoster; *P*, *P*‐value; PHN, postherpetic neuralgia; PY, person‐years; y, years. ^†^aIRRs were calculated using generalized linear models assuming a negative binomial distribution and log link, accounting for the propensity score of being diagnosed with COPD and relevant demographic characteristics at incident HZ (i.e., age, sex, region and insurance type) and clinical characteristics before incident HZ (i.e., CCI score; asthma; use of chemotherapy, immunosuppressants and corticosteroids [short‐term oral, long‐term oral and inhaled]). aIRRs per age group are not reported as the sample sizes were not sufficient to estimate stable aIRRs by age group. ^‡^For the numbers of patients in each group, please refer to Table S3. ^§^The mean overall observation periods were 21.2 ± 17.1 months in the COPD+/HZ+ cohort and 23.9 ± 17.1 months in the COPD−/HZ + cohort, with similar periods by age group.

The incidence rate of PHN was higher in the COPD+/HZ+ versus COPD−/HZ+ cohorts (64.8 vs. 37.1 per 1000 PY) (Figure 4). However, after adjusting for demographic and clinical characteristics, there was no significant difference between the cohorts (aIRR, 1.07; 95% CI, 0.79 to 1.45; *P* = 0.65). Nevertheless, unadjusted PHN incidence rates were higher among those in the COPD+/HZ+ versus COPD−/HZ+ cohort in all age groups (Figure 4). Further, PHN incidence rates increased with age in both cohorts, from 29.9 to 76.7 per 1000 PY in the COPD+/HZ+ cohort and from 15.3 to 60.2 per 1000 PY in the COPD−/HZ+ cohort for age groups of 40–49 through ≥80 years (Figure 4).

## 4 DISCUSSION

In this large US database study of people aged ≥40 years, the incidence rate of HZ was higher among patients with versus without COPD (13.0 vs. 2.3 per 1000 PY). After adjustment for baseline demographic and clinical characteristics, those with versus without COPD had a 2.8‐fold higher incidence rate of HZ (*P* < 0.001). The incidence rates of HZ increased with age in the COPD+ and COPD− cohorts. aIRRs were similar across age groups, with higher incidence rates of HZ in the COPD+ versus COPD− cohorts in each age group. Overall, PHN was more common in the COPD+/HZ+ versus COPD−/HZ+ cohort, but after adjustment for baseline characteristics, there was little difference between the two cohorts.

Our overall incidence rate of HZ in the COPD+ cohort (13.0 per 1000 PY [age ≥40 years; 2013–2018]) is in line with previous studies that included COPD patients from the US (11.4 per 1000 PY [≥65 years; 2007–2009]),^12^ the United Kingdom (5.6–11.5 per 1000 PY [≥50 years; 2000–2011]),^13^ Spain (11.4 per 1000 PY [≥18 years; 2009–2012]),^14^ Germany (8.7 per 1000 PY [≥18 years; 2018])^17^ and Taiwan (16.4 per 1000 PY [≥50 years; 2005]).^10^

Our incidence rate of HZ in the COPD− cohort (1.7–3.0 per 1000 PY with increasing age) is lower than in a recent general US population study (5.0–12.0 per 1000 PY among similar age groups [2007–2018]).^4^ This may be a reflection of the particularly healthy status of our COPD− population after the exclusion of patients with COPD, which resulted in a population with very few comorbidities, as shown by their considerably lower CCI score than the COPD+ population (0.2 vs. 1.9). For example, only 3.4% of the COPD− population had diabetes, 1.5% heart failure and 0.7% asthma, compared to rates of approximately 11–21% for diabetes,^20^ 1–6% for heart failure^21^ and 9% for asthma^22^ among US adults of generally similar ages. This could be partly because our baseline observation period was only 6 months, but rates in the COPD+ cohort were in line with (or considerably higher than) these other rates despite a similarly short baseline (diabetes: 18.5%; heart failure: 20.6%; asthma: 16.2%).

Despite this, our result that people with COPD are at increased risk of HZ is in line with previous studies from the US,^11^, ^12^, ^15^ the United Kingdom,^13^ Spain,^14^, ^16^ Germany^17^ and Taiwan^10^ and two meta‐analyses.^23^, ^24^ However, while these studies reported a 1.1–1.7‐fold increased risk of HZ among those with COPD,^10^, ^11^, ^12^, ^13^, ^14^, ^15^, ^16^, ^17^, ^23^, ^24^ we found a 2.8‐fold higher risk, likely due to the low observed incidence rate of HZ in the COPD− cohort. This increased risk has been suggested to be due to chronic inflammation and immune system impairment/dysregulation in patients with COPD.^10^, ^16^

In the current study, we adjusted for important differences between the COPD+ and COPD– cohorts, including age, comorbidities and use of corticosteroids, all of which were considerably higher in the COPD+ cohort. As in the current study, increasing age has been associated with an increased risk of HZ, both among people with COPD^10^, ^13^, ^16^ and among those without,^4^, ^13^, ^14^, ^16^ likely due to the natural decline in immunity as a person ages.^2^, ^3^ Various comorbidities have also been associated with an increased risk of HZ, potentially related to immunosuppression and autoimmunity.^23^, ^24^ Likewise, use of corticosteroids has been linked to an increased risk of HZ, again, likely due to immunosuppression.^25^ Interestingly, a Spanish study reported the incidence rates of HZ among patients with COPD who were and were not taking inhaled corticosteroids versus those without COPD (13.0 and 11.1 vs. 7.0 per 1000 PY, respectively).^16^ Similarly, a study from Taiwan reported higher incidences of HZ for patients with COPD who were taking oral or inhaled corticosteroids (26.3 and 18.4 per 1000 PY, respectively) compared to those with COPD who were not taking corticosteroids (14.3 per 1000 PY) and people without COPD (8.8 per 1000 PY).^10^ These data^10^, ^16^ indicate that COPD has an impact on HZ risk above and beyond corticosteroid use, but that the risk increases among patients with COPD who also require corticosteroids.

The incidence rate of PHN has been shown to increase with age in the general population,^4^, ^26^ and this was also the case in the COPD−/HZ+ cohort in the current study. We additionally showed that the incidence rate of PHN increased with age among those in the COPD+/HZ+ cohort, a finding that has, to our knowledge, not yet been reported. We also found that the overall incidence rate of PHN was higher in the COPD+/HZ+ versus COPD−/HZ+ cohorts (64.8 vs. 37.1 per 1000 PY), but this increased risk was not maintained after adjustment for baseline characteristics. A Spanish study has also reported an increased risk of PHN among patients with versus without COPD, although their increased risk remained after adjustment.^26^ To our knowledge, no other studies have examined the risk of PHN by COPD status, hence these somewhat disparate results need to be confirmed in future studies.

The main strengths of the current study are that we included a large sample size and adjusted for demographic factors, asthma, comorbidities and medication use when comparing HZ incidence rates between the COPD+ and COPD− cohorts. However, the data in Optum CDM are primarily generated for the payment of health services, so did not include certain clinical variables, including pulmonary function tests, physician notes and some patient characteristics (e.g., smoking status and body mass index). Also, use of a single claim associated with an HZ diagnosis may result in an inaccurate estimation of the incidence of HZ. In addition, it is likely that some people had undiagnosed COPD, particularly those with less severe disease.

Approximately 50% of the population had Medicare Advantage coverage, with the remainder having Commercial coverage. Although Optum CDM covers approximately 13 million annual lives of adults in the US, the study results may, therefore, not be generalizable to other populations (e.g., those covered by Medicaid), particularly considering the low incidence rate of HZ in the COPD− cohort, which could indicate a healthier‐than‐normal population.

Although we took steps to exclude patients who had received HZ vaccination (due to their reduced risk for HZ), this only accounted for 4.1% and 1.0% of the COPD+ and COPD– cohorts, respectively (Figure 2), whereas HZ vaccination coverage was considerably higher than this in the US at the time of the current study (25–35% among those aged ≥60 years).^27^ It is therefore possible that some patients could have received HZ vaccination before the start of the baseline period, which could partly explain the low incidence rates of HZ in the current study. However, some patients would have received HZ vaccination during follow‐up, at which point they would have been censored. We also note that the HZ vaccination recommendations changed (from ZVL for those aged ≥60 years^6^ to RZV for those aged ≥50 years^7^) towards the end of the study, so 17% and 47% of the COPD+ and COPD− cohorts, respectively, would not have been eligible for vaccination for most of the study period. Certainly, future studies will have to consider the potential impact of differential and increasing HZ vaccination rates.

Our results confirm that adults with COPD have a significantly increased risk of developing acute HZ, even after adjustment for factors such as age, comorbidities and corticosteroid use. Results further confirm that the risk of HZ increases with age, among people with and without COPD. These results may help to strengthen efforts for HZ prevention (e.g., vaccination) from health care professionals who care for people with COPD.

## CONFLICT OF INTEREST

P.T.L., W.Y.C. and M.S.D. are employees of Analysis Group, Inc., a consultancy that received funding from GSK to conduct this study. M.J.W. and S.P. were employees of Analysis Group, Inc. at the time of study conduct and manuscript development. M.J.W. is now employed by, and holds shares in, Amgen Inc. S.P. is now employed by Takeda. P.G. and M.B. were employed by GSK at the time of study conduct and manuscript development and holds shares in GSK. P.G. is now employed by, and holds shares in, Moderna. M.B. is now employed by Gilead Sciences. B.J.P. was employed by, and held shares in, GSK at the time of study conduct. B.J.P. is currently employed by Janssen Global Services LL and holds shares in Johnson & Johnson. B.P.Y. reports having received personal fees from GSK for consultation related to chronic obstructive pulmonary disease (COPD) and herpes zoster (HZ) during the conduct of this study. B.P.Y. also reports a research grant from GSK to her institution for unrelated study of COPD and HZ and reports having received personal fees from AstraZeneca, Boehringer Ingelheim, GSK, Novartis and TEVA for participation in advisory boards related to COPD and HZ, and COPD only, outside the present study. The authors declare no other financial and non‐financial relationships and activities.

## ETHICS STATEMENT

The study employed a retrospective longitudinal cohort study design using a large administrative claims database. Data were de‐identified and comply with the requirements of the Health Insurance Portability and Accountability Act. Institutional Review Board review and approval was therefore not required, as per United States Department of Health and Human Services (HHS) regulation for the protection of human subjects in research (45 CFR 46, www.hhs.gov/ohrp/regulations-and-policy/regulations/45-cfr-46). The study has been conducted in accordance with the guiding principles of the Declaration of Helsinki. As only existing de‐identified data have been analyzed and as patients have not been contacted during the course of this study, informed consent process is not applicable.

## AUTHOR CONTRIBUTIONS

All authors participated in the design or implementation or analysis, and interpretation of the study, and in the development of this manuscript and in its critical review with important intellectual contributions. All authors had full access to the data and gave approval before submission. All authors agreed to be accountable for all aspects of the work in ensuring that questions related to the accuracy or integrity of any part of the work are appropriately investigated and resolved. The work described was carried out in accordance with the recommendations of the International Committee of Medical Journal Editors for conduct, reporting, editing and publication of scholarly work in medical journals.

## Supporting information


**Table S1.** Definitions
**Table S2.** Baseline CCI data
**Table S3.** Baseline demographic and clinical characteristics of patients with incident HZ

**Video S1.** Supporting information

## Data Availability

GSK makes available anonymized individual participant data and associated documents from interventional clinical studies which evaluate medicines (upon approval of proposals submitted to www.clinicalstudydatarequest.com ). To access data for other types of GSK sponsored research, for study documents without patient‐level data and for clinical studies not listed, please submit an enquiry via the website.
