# Supplementary material for: Chronic obstructive pulmonary disease is associated with an increased risk of herpes zoster: A retrospective United States claims database analysis
Source: Clin Respir J. 2022 Nov 22;16(12):826–34. doi: 10.1111/crj.13554 (PMC9716712; doi:10.1111/crj.13554)
Supplement: Supplementary file 1 — Table S1. Definitions Table S2. Baseline CCI data Table S3. Baseline demographic and clinical characteristics of patients with incident HZ [file CRJ-16-826-s001.pdf]

Supplementary Text 1. Standardized difference calculations

- Standardized differences of continuous variables were calculated as the absolute difference in the means divided by the square root of the average of the squared standard deviations.
- Standardized differences of categorical variables were calculated as $(P_1-P_2)/\sqrt{p(1-p)}$, where P_1 = the proportion of participants in the COPD+ cohort, P_2 = the proportion of participants in COPD– cohort, and $p = (P_1+P_2)/2$.

Supplementary Table 1. Definitions

Diagnosis	Definition
COPD (modified from Dalal et al[1])	≥1 claim for an inpatient stay with COPD as a primary diagnosis (ICD-9-CM: 491, 492, 496; ICD-10-CM: J41, J42, J43, J44)<br>• ≥1 claim for an outpatient visit with COPD as a primary diagnosis and ≥1 outpatient visit with a diagnosis of COPD (in any position) on a separate date within 12 months<br>• ≥1 claim with a diagnosis of COPD (in any position) and a filled prescription for a COPD maintenance treatment within 12 months (LAMA, LABA, ICS/LABA, LABA/LAMA, ICS/LABA/LAMA)
HZ	≥1 claim associated with a diagnosis of HZ (ICD-9-CM: 053 excluding 053.1; ICD-10-CM: B02 excluding B02.2)
PHN	≥1 claim associated with a diagnosis of PHN (ICD-9-CM: 053.1; ICD-10-CM: B02.2) and ≥1 claim for a medication or pain intervention ≥90 days after the PHN or HZ claim
HZO	• ≥1 claim associated with a diagnosis of HZO (ICD-9-CM: 053.21, 053.22, 053.29; ICD-10-CM: B02.31, B02.32, B02.33, B02.34, B02.39)<br>• ≥1 claim associated with a diagnosis of eye complications within 30 days of an HZ diagnosis (ICD-9-CM: 363.20, 364.3, 369, 370, 379.0, 379.43; ICD-10-CM: H15.0, H15.1, H16, H20.9, H30.93, H54, H57.04)
Asthma	≥1 claim associated with an asthma diagnosis (ICD-9-CM: 493; ICD-10-CM: J45)

COPD, chronic obstructive pulmonary disease; HZ, herpes zoster; HZO, herpes zoster ophthalmicus; ICD-9-CM, International Classification of Diseases, Ninth Revision, Clinical Modification; ICD-10-CM, International Classification of Diseases, Tenth Revision, Clinical Modification; ICS, inhaled corticosteroids; LABA, long-acting beta agonist; LAMA, long-acting muscarinic antagonist; PHN, postherpetic neuralgia.

Reference

1. Dalal AA, Liu F, Riedel AA. Cost trends among commercially insured and Medicare Advantage-insured patients with chronic obstructive pulmonary disease: 2006 through 2009. *Int J Chron Obstruct Pulmon Dis*. 2011;6:533–542.

Supplementary Table 2. Baseline CCI data

	COPD+ cohort (n=161,970)	COPD– cohort (n=9,643,522)	Standardized difference†
CCI score,‡ n (%)			
0	30146 (18.6)	8953790 (92.8)	149.5%
1	62277 (38.4)	244460 (2.5)	89.0%
2-4	54140 (33.4)	387228 (4.0)	75.4%
≥5	15407 (9.5)	58044 (0.6)	40.7%
CCI conditions, n (%)			
Chronic pulmonary disease§	116860 (72.1)	176176 (1.8)	145.7%
Congestive heart failure	33293 (20.6)	149255 (1.5)	60.6%
Diabetes without chronic complications	29990 (18.5)	330558 (3.4)	48.3%
Peripheral vascular disease	26968 (16.6)	117387 (1.2)	54.1%
Renal disease	23242 (14.3)	147683 (1.5)	47.4%
Cerebrovascular disease	18487 (11.4)	138683 (1.4)	40.7%
Diabetes with chronic complications	17582 (10.9)	128350 (1.3)	39.8%
Any malignancy¶	15935 (9.8)	138489 (1.4)	36.4%
Myocardial infarction	11756 (7.3)	72995 (0.8)	33.1%
Mild liver disease	6239 (3.9)	51409 (0.5)	22.7%
Rheumatologic disease	6389 (3.9)	49259 (0.5)	23.3%
Dementia	5612 (3.5)	55656 (0.6)	20.5%
Metastatic solid tumor	2980 (1.8)	29252 (0.3)	14.9%
Peptic ulcer disease	2522 (1.6)	19414 (0.2)	14.5%
Hemiplegia or paraplegia	1975 (1.2)	23111 (0.2)	11.5%
Moderate or severe liver disease	853 (0.5)	7231 (0.1)	8.2%
AIDS/HIV	357 (0.2)	5332 (0.1)	4.5%

AIDS, acquired immune deficiency syndrome; CCI, Charlson-Quan Comorbidity Index; COPD, chronic obstructive pulmonary disease; HIV, human immunodeficiency virus; n, number of patients.

†Standardized differences of 20%, 50%, and 80% suggest small, medium, and large differences, respectively.[1]

‡Computed according to the methods outlined in Quan et al.[2]

§Chronic pulmonary disease included COPD, pulmonary heart disease, bronchitis, asthma, bronchiectasis, pneumoconiosis, etc. As the index date was the later of the first observed COPD diagnosis or 6 months after the start of continuous enrollment, not all patients in the COPD+ cohort had this in their baseline period.

¶Including leukemia and lymphoma, but not malignant neoplasms of the skin.

References

1. Cohen J. Statistical power analysis for the behavioral sciences. 2nd ed. Mahwah (NJ): Lawrence Erlbaum Associates; 1988.
2. Quan H, Li B, Couris CM, et al. Updating and validating the Charlson comorbidity index and score for risk adjustment in hospital discharge abstracts using data from 6 countries. *Am J Epidemiol*. 2011;173(6):676–682.

Supplementary Table 3. Baseline demographic and clinical characteristics of patients with incident HZ

	COPD+/HZ+ cohort (n=6430)	COPD–/HZ+ cohort (n=69 524)	Standardized difference†
Age at incident HZ (y), mean±SD	73.0±9.6	65.9±12.5	63.8%
40-49 y, n (%)	109 (1.7)	8974 (12.9)	43.1%
50-59 y, n (%)	628 (9.8)	16 167 (23.3)	36.3%
60-69 y, n (%)	1419 (22.1)	16 120 (23.2)	2.7%
70-79 y, n (%)	2611 (40.6)	17 268 (24.8)	33.6%
≥80 y, n (%)	1663 (25.9)	10 995 (15.8)	24.7%
Male, n (%)	2539 (39.5)	26 188 (37.7)	3.7%
Race/ethnicity, n (%)			
White	4271 (66.4)	44 409 (63.9)	5.3%
Black	523 (8.1)	4582 (6.6)	5.9%
Hispanic	430 (6.7)	6245 (9.0)	8.5%
Asian	144 (2.2)	2311 (3.3)	6.6%
Unknown	1062 (16.5)	11 977 (17.2)	1.9%
Geographic region, n (%)			
South	2589 (40.3)	26 885 (38.7)	3.3%
West	1617 (25.1)	16 847 (24.2)	2.1%
Midwest	1420 (22.1)	17 350 (25.0)	6.8%
Northeast	781 (12.1)	8226 (11.8)	1.0%
Unknown	23 (0.4)	216 (0.3)	0.8%
Insurance type, n (%)			
Medicare Advantage	5592 (87.0)	37 525 (54.0)	72.3%
Commercial	838 (13.0)	31 999 (46.0)	–
Asthma, n (%)	1190 (18.5)	2809 (4.0)	45.7%
CCI score,‡ mean±SD	2.2±2.0	0.7±1.4	89.5%
Use of oral corticosteroids, n (%)	2811 (43.7)	14 764 (21.2)	48.0%
Short-term use§	2337 (83.1)	13 465 (91.2)	24.1%
Long-term use¶	474 (16.9)	1299 (8.8)	–
Use of inhaled corticosteroids, n (%)	2124 (33.0)	1445 (2.1)	81.4%
Use of immunosuppressants, n (%)	80 (1.2)	574 (0.8)	4.1%

CCI, Charlson-Quan Comorbidity Index; COPD, chronic obstructive pulmonary disease; HZ, herpes zoster; n, number of patients; SD, standard deviation; y, years.

†Standardized differences of 20%, 50%, and 80% suggest small, medium, and large differences, respectively.[1]

‡Computed according to the methods outlined in Quan et al.[2]

§<6 consecutive weeks.

¶≥6 consecutive weeks, allowing for up to 7 days of gap between two dispensing.

References

1. Cohen J. Statistical power analysis for the behavioral sciences. 2nd ed. Mahwah (NJ): Lawrence Erlbaum Associates; 1988.
2. Quan H, Li B, Couris CM, et al. Updating and validating the Charlson comorbidity index and score for risk adjustment in hospital discharge abstracts using data from 6 countries. *Am J Epidemiol*. 2011;173(6):676–682.
